# Cycloid Psychosis: Navigating the Nosological Grey Zone – A Case Report

**DOI:** 10.1192/j.eurpsy.2025.2250

**Published:** 2025-08-26

**Authors:** C. Portela, R. Dionísio, S. M. Sousa, D. Areias, M. Santos, B. Baptista

**Affiliations:** 1 ULS Santo António, Porto, Portugal

## Abstract

**Introduction:**

Cycloid psychosis, first described by Kleist and further developed by Leonhard, is characterized by a cyclic course of acute psychotic episodes, marked by delusions, hallucinations, affective and motor disturbances. It lies at the intersection of affective and schizophreniform disorders, presenting a diagnostic challenge due to its episodic nature and inter-episodic remission. Currently, no diagnostic category clearly encompasses such clinical pictures, and these patients are usually diagnosed with brief psychotic disorders.

**Objectives:**

This report aims to describe a case of cycloid psychosis, highlighting the nosological and treatment limitations of this entity.

**Methods:**

Consultation and review of the patient’s clinical records and articles referenced on the PubMed platform.

**Results:**

This case report presents a 37-year-old male admitted to the emergency department with delusions of persecution and reference, auditory hallucinations, decreased need for sleep, and increased goal-directed activity, alongside a lack of insight into his condition. No significant mood disturbances were observed. Symptoms had progressively worsened over two weeks, and upon examination, the patient exhibited psychomotor agitation, incoherent speech, perplexity, and marked distress. Initial laboratory work and brain imaging were unremarkable. The patient was hospitalized for involuntary treatment, where antipsychotic therapy with risperidone (up to 4mg/day) was initiated but later switched to olanzapine (10mg/day) due to significant extrapyramidal symptoms. He was also started on valproic acid, titrated up to 1000mg/day. During hospitalization, the patient showed progressive behavioral organization, resolution of delusions and auditory hallucinations, improved sleep, and restored insight.

Upon review of his psychiatric history, it was discovered that he had experienced two previous psychotic episodes in 2018 and 2021, diagnosed as *bouffées délirantes* while residing in France. Both episodes were successfully treated with olanzapine, with *restituto ad integrum* within one month, and no signs of personality changes or biographical disruption. Based on these recurrent psychotic episodes and his current presentation, a diagnosis of cycloid psychosis was made, following the criteria proposed by Perris and Brockington.

The patient was discharged after two weeks of inpatient care, and at his one-week follow-up, showed complete remission of psychotic symptoms.

**Conclusions:**

Cycloid psychosis presents significant diagnostic challenges due to its ambiguous nosological status. It does not fit neatly into conventional categories such as schizophrenia or bipolar disorder, as it shares characteristics with both while maintaining a distinct clinical course marked by episodic, self-limiting psychotic phases with full remission. This diagnostic ambiguity also poses difficulties in treatment, as no specific guidelines exist, and current literature is sparce.

**Disclosure of Interest:**

None Declared

